# Cost and value in medical education: the factor of change management

**DOI:** 10.11604/pamj.2016.24.246.6841

**Published:** 2016-07-15

**Authors:** Kieran Walsh

**Affiliations:** 1 BMJ Learning, London; 2 BMJ Publishing Group, London

**Keywords:** Medical education, cost, economics

## Editorial

Medical education is expensive. The cost of medical education must cover undergraduate education, postgraduate training and continuing professional development. The cost of all these components has led to a growing interest in how to develop cost effective or high value forms of medical education [1]. This interest has resulted in some initiatives whereby costs could be saved and value delivered [2–4]. However even if a new form of low cost high value medical education is developed, consideration must be given to the change management process whereby the old system is gradually retired and the new one taken up. Consideration must also be given to the cost of the change management process.

These costs can be considerable and could even outweigh any savings that might result from the new system of medical education. Even though the costs of change management are by their nature short term they can cause stakeholders to question the entire process and to wonder whether the whole project is worthwhile. These concerns will be particularly telling when one of the core purposes of the process is to save costs. Stakeholders will simply say that any saved costs will be wasted on a long and expensive change management process. For these reasons it vital that attention is paid to the costs of the change management process and that its costs are strictly controlled. According to Richard Hooker, “change is not made without inconvenience, even from worse to better” [5]. This is certainly true of change in medical education.

When planning a change, it is most important first of all to establish the costs. If the costs are not known in the first place it will be difficult to control them or to ensure that they do not overrun. The costs of change management are largely accounted for by people costs. People are required at all stages of the change management process - from identifying barriers to change, to understanding the barriers to change, to overcoming the barriers to change [6]. The people involved whose costs must be accounted for will include the core project ream who are managing the change and the faculty who deliver the education. The project team will likely be spending all of their time on the change process - albeit for a limited amount of time - but the rest of the faculty will be spending only some of their time on it. Nonetheless it is important that all faculties are engaged to a greater or lesser extent.

What is the most cost efficient way to undertake the first step - identifying barriers to change? Talking to important stakeholders is an effective way of identifying barriers. It is certainly low cost and does not take much time [7]. It also enables the change management team to act and learn in an iterative manner and as a corollary of this in an efficient manner. Another way to identify barriers is to simply observing educational practice in action. Once again this can be almost immediate and the only resource required is the time of the change management team. Using a questionnaire to seek out the thoughts of a wider range of stakeholders is also sensible. Many online questionnaire programmes are now freely available. The online software can also enable rapid analysis of the results of the questionnaire. However time and thus cost must be spent on the design of questions for the questionnaire. The quality of the answers that will be gleaned from the questionnaire will be heavily dependent on the questions asked. Running a focus group is another way of identifying barriers to change. However this method takes both time and resources. A facilitator will be required to run the group; the time of a number of different staff will be taken up; and experts will be required to analyse the outcomes from the groups. To identify all the barriers to change it is best to use more than a single method - multiple methods will be more likely to identify all potential barriers. This will increase the cost but should be worthwhile in the long term. An iterative approach to combining the methods is often wise - hypotheses regarding barriers identified by questionnaire can subsequently be tested on focus groups.

The next step is understanding the identified barriers to change [8]. A variety of barriers to change can exist - these might be related to knowledge, skills, attitudes, motivation, practicalities, or even barriers that are completely outside our control. The specific barriers identified will depend on the exact change management process that is happening. The important thing is to spend a modest amount of time and thus resource in understanding the barriers and categorising them. The most important categorisation is establishing which barriers are in the team's power to change and which are not. It is an inefficient and wasteful use of resources to spending time thinking about barriers that cannot be overcome. It is also inefficient to think about knowledge transfer as a means of overcoming a barrier when in fact there is no knowledge deficit but rather a sometimes understandable lack of motivation to change.

The next and final step is overcoming the barriers. There is no single best way to do this and sometimes it is most effective and most efficient to use a range of methods. It is often most efficient to tailor the method of change management to the particular barrier that needs to be overcome. Using educational materials is one way to overcome barriers to change. Educational materials are most likely to be helpful when the barrier relates to a knowledge or skill gap. Educational materials can be low cost - however they will rarely work on their own - largely because it is relatively rare that an educational deficit alone constitutes the only barrier to change. Educational meeting are another way of overcoming barriers. Once again they will most likely help when it is predominantly an educational barrier that needs to be overcome. However an important caveat is that meetings are expensive. Large scale traditional meetings are expensive because of their size, and small interactive meetings are expensive because of the increased investment required to make the interactivity high quality. Educational meetings might work but they are likely to be costly. Educational outreach visits are an alternative means of overcoming barriers to change. They are certainly effective in helping to change practice. However they are costly and take time to implement. Using this method of change management is likely to raise questions as to whether the means used to overcome a problem are more problematic than the problem itself. Another approach is to use quality improvement methodology to drive forward the change [9]. This might involve using plan-do-study-adjust cycles and continual re-measurement to implement change. This can be expensive - however there is growing evidence of its effectiveness. Depending on the outcome to be achieved it may turn out to be worth the expense.
